# Increased inflammation and endothelial markers in patients with late severe post-thrombotic syndrome

**DOI:** 10.1371/journal.pone.0227150

**Published:** 2020-01-16

**Authors:** Luis Fernando Bittar, Letícia Queiroz da Silva, Fernanda Loureiro de Andrade Orsi, Kiara Cristina Senger Zapponi, Bruna de Moraes Mazetto, Erich Vinícius de Paula, Silmara Aparecida de Lima Montalvão, Joyce Maria Annichino-Bizzacchi

**Affiliations:** Hematology and Hemotherapy Center, University of Campinas, Campinas, São Paulo, Brazil; Universite de Liege (B34), BELGIUM

## Abstract

**Introduction:**

Post-thrombotic syndrome (PTS) is a limiting long-term complication present in 20–50% of patients with deep venous thrombosis (DVT) of the lower limbs. A panel of biomarkers with potential relevance to enhance knowledge on the pathophysiology of PTS was investigated.

**Methods:**

This case-control study included 93 patients with DVT in the lower limbs, 31 with severe PTS (cases) and 62 with mild/no PTS (controls), over 24 months after an acute episode. Thirty-one healthy individuals (HI) with no history of DVT were included as a reference to the population. FVIII activity, D-dimer, inflammatory cytokines, endothelial dysfunction markers, matrix metalloproteinases, and their inhibitors, tissue remodeling and growth factor levels were evaluated. The classification of PTS was, by the Villalta scale.

**Results:**

Patients with severe PTS showed elevated levels of CRP, sICAM-1, sE-selectin, and decreased MMP-9 and MCP-1 levels when compared to patients with mild/no PTS. Moreover, DVT patients presented higher levels of FVIII and D-dimer when compared to HI.

**Conclusions:**

DVT patients present an inflammatory status, endothelial dysfunction and altered proteolysis MMPs activity, even a long time after the acute thrombotic episode, which is more significant in severe PTS. These results suggest a possible role of these mediators in the maintenance and worsening of PTS severity.

## Introduction

Post-thrombotic syndrome (PTS) is a long-term complication present in 20–50% of patients with deep venous thrombosis (DVT) of the lower limbs, even when optimal anticoagulant therapy is used to treat the thrombotic episode [1,2]. Patients present clinical symptoms in the lower limb as pain, heaviness, itching, cramps, and tingling, which can be graded from mild to intense complaints during daily activities, and severe PTS can be accompanied by chronic venous leg ulceration [3]. PTS is associated with morbidity, poor quality of life, and a significant cost to the healthcare system. Furthermore, severe PTS occurs in 5–10% of patients with DVT of the lower limbs, these present quality of life compared to patients with heart failure or cancer [4,5].

The etiopathogeny of PTS has not yet been entirely understood. Venous hypertension seems to play a central role in the clinical presentation of PTS, as a result of chronic inflammation, decreased fibrinolysis and vein obstruction, tissue remodeling, and endothelial activation[6–8]. Upon the occurrence of DVT, endothelial cells are activated in response to endothelial injury, and this activation results in increased surface expression of cell adhesion molecules (CAMs), such as P-selectin, E-selectin, vascular cell adhesion molecule 1 (VCAM-1), and intercellular adhesion molecule 1 (ICAM-1), promoting adhesion and activation of leukocytes to the endothelium, amplifying thrombosis and inflammation [9,10]. Thus, the presence of growth factors, proteases, and cytokines secreted by leukocytes damage venous valves, provoking reflux and venous hypertension [11,12]. Wall fibrosis is a result of fibroblasts and smooth muscle cells remodeling and collagen deposition [13]. Previous studies have suggested that matrix metalloproteases (MMPs) are involved in tissue remodeling after DVT, also contributing to post-thrombotic venous wall damage [14–17]. However, except for inflammation, very few studies have investigated these pathways in patients with PTS. Thus, we performed a case-control study including patients with severe, mild and without PTS to investigate the potential relevance of biomarkers that could be involved in the pathophysiology of this DVT complication.

## Design and methods

### Study population

In compliance with the Declaration of Helsinki, experimental procedures were approved by the local Ethics Committee of the University of Campinas on Human Research, and written informed consent was obtained from all study participants (process Nº 841.389).

This case-control study included patients with at least one episode of DVT of the lower limbs attended at the Hemostasis and Thrombosis outpatient clinic of a State University, between January 2012 and May 2015. Inclusion criteria were symptomatic and objectively confirmed DVT of the lower limbs, treated with anticoagulants for at least 3 months. Time elapsed since the first DVT should be lower than 24 months.

From 500 consecutive adult patients attended at the clinic after anticoagulant treatment for symptomatic DVT, 252 could not be included in the study due to exclusion criteria. Reasons for exclusion were: DVT of other sites (N = 154), under 18 years of age (N = 38), history of cancer < 5 years (N = 20), infection, liver or renal disease (N = 40). All acute episodes of DVT were confirmed by duplex ultrasonography. DVT episodes were classified as unprovoked or provoked when the episode occurred in the presence of acquired risk factors such as surgery, immobilization, pregnancy/puerperium, or use of contraceptives. Inherited risk factors were not taken into consideration.

From the 248 patients considered for participation, 31 patients with a history of DVT and with the Villalta scale ≥ 15 points were selected as cases. After the selection of this group, we carefully chose control group, which comprised 31 patients with DVT/ mild PTS and 31 patients DVT/ without PTS. Given that the clinical condition of DVT with mild or without PTS is very similar we considered both as a single group. In addition, 31 healthy individuals (HI) without DVT were included and matched by age and gender. This former group was from the same geographical area and exclusion criteria were the same as for the patients, including personal and familial history for DVT.

### Diagnosis and classification of PTS

All patients were examined by the same investigator, and the clinical evaluation of PTS was performed on the same day as blood collection. The presence and severity of PTS were classified according to the Villalta scale [18]. Points were given for symptoms and clinical signs, ranging from ≤ 0 (no PTS) to ≥ 15 points (severe PTS).

### Laboratory methods

After an overnight fast, venous blood samples (30 mL) were collected from all participants, drawn from the antecubital vein into Vacuette^®^ tubes (Greiner Bio-One, Austria): 0.129 mmol/L trisodium citrate tube, ethylenediaminetetraacetic (EDTA) tube, and Z Serum Sep Clot Activator tube. Samples were immediately centrifuged for 20 minutes at 1500 *g*, and plasma/serum was directly frozen and stored at -80°C for an average of 15 months (6 months minimum and 24 months maximum). Any re-frozen event was reported., FVIII activity was measured by a one-stage clotting assay with FVIII-deficient plasma (Siemens, Marburg, Germany) as recommended by the manufacturer. FVIII tests were performed in duplicate on an automated coagulation analyzer (BCS XP, Siemens, Marburg, Germany). Laboratory normal range was 62.0 to 151.0 IU/dL. D-dimer plasma levels were performed by immunoturbidimetric analysis, as recommended by the manufacturer, on an automated coagulation analyzer (BCS XP, Siemens, Marburg, Germany). Normal laboratory values were considered levels at ≤ 0.55 mg/L. ABO blood group was determined by agglutination and adsorption-elution test, PK-7200 (Olympus). Serum C Reactive Protein (hs-CRP) levels were determined by a nephelometric method (Siemens, Marburg, Germany), on Siemens BN ProSpec analyzer. Normal range was considered < 0.50 mg/dL.

#### Inflammatory cytokines, endothelial dysfunction markers, matrix metalloproteinases, and their inhibitors, tissue remodeling and growth factors levels by multiplex analysis

The Milliplex Multiplex Assay (Millipore, EUA) is a magnetic bead-based multiplex assay that enables the simultaneous measurement of several protein biomarkers. The assay was performed as per manufacturer’s instructions. All samples were acquired on a Bioplex-200 instrument (Bio-Rad, Marnes-la-Coquette, France). Biomarkers analyzed by Milliplex Multiplex Assay were: soluble tissue factor (s-TF), thrombomodulin, interleukin 1 β (IL-1β), interleukin 6 (IL-6), interleukin 8 (IL-8), interleukin 10 (IL-10), interleukin 13 (IL-13), monocyte chemoattractant protein 1 (MCP-1), tumor necrosis factor-alpha (TNF-α), RANTES, Endothelin-1, soluble E-selectin (sE-selectin), soluble P-selectin (sP-selectin), soluble intercellular adhesion molecule 1 (sICAM-1), soluble vascular cell adhesion molecule 1 (sVCAM-1), matrix metallopeptidase 1 (MMP-1), MMP-2, MMP-3, MMP-7, MMP-9, MMP-10, MMP-12, MMP-13, tissue inhibitor of metalloproteinases 1 (TIMP-1), tissue inhibitor of metalloproteinases 2 (TIMP-2), platelet-derived growth factor-AA (PDGF-AA), platelet-derived growth factor-AB/BB (PDGF-AB/BB), epidermal growth factor (EGF), fibroblast growth factor 1 (FGF-1), fibroblast growth factor 2 (FGF-2), transforming growth factor-beta 1 (TGF- β1), transforming growth factor-beta 2 (TGF- β2), transforming growth factor-beta 3 (TGF- β3).

### Statistical analysis

Categorical variables were described as number and percentage and continuous variables were described as mean and standard deviation (SD). Clinical characteristics were compared using Fisher’s exact test or Mann-Whitney test. The association between PTS and the pre-specified biomarkers was evaluated using general linear models adjusted for age and gender. Further adjustments for multiple comparisons were performed using the Bonferroni test. A P value < 0.05 was considered statistically significant. All analyses were performed using the R Foundation for Statistical Computing, version 3.2.4.

Since sICAM-1 levels have been previously associated with PTS risk [19,20], for the statistical power measurement, the primary endpoint was defined as the difference in sICAM-1 level between no/mild PTS and severe PTS groups. In the no/mild PTS group, we observed that the mean (±SD) of the sICAM-1 levels was 55.2 (±16.9) ng/mL. Therefore, we expected a-priori to find a mean difference of at least 10 ng/mL between the control and case group with a 2-sided alpha of 0.05 and 80% of statistical power.

## Results

### Study population

Initially, thirty-one patients with PTS ≥ 15 points were included as cases, 8 male and 23 female patients. Additionally, 62 DVT patients with PTS score below 9 points or without PTS were included as controls, 16 male and 46 females. The baseline characteristics of the study population are shown in Table 1. There was no significant difference between the groups related to age, gender, ABO blood group, body mass index (BMI) and time between last DVT and study inclusion. DVT was spontaneous in 33 (35.5%) patients. In the remaining 60 patients (64.5%), DVT-associated risk factors included use of hormonal contraceptive (n = 19), antiphospholipid antibody syndrome (n = 6), surgery (n = 11), immobilization (n = 7), pregnancy or puerperium (n = 9), trauma (n = 5), cancer > 5 years (n = 2) and thrombophlebitis (n = 1). Regarding the DVT localization, 84/93 (90.3%) were proximal DVT and 9/93 (9.7%) were distal DVT. Importantly, 32/93 (34.4%) of DVT patients had a history of prior DVT episodes (recurrent DVT).

**Table 1:** Baseline characteristics of DVT patients.

	Total Patients (N = 93)	HI (N = 31)	P*	No / Mild PTS (N = 62)	Severe PTS (N = 31)	P**
**Age** *Mean (SD)*	49.7 (11.2)	49.5 (11.4)	0.96	49.3 (11.1)	50.4 (11.5)	0.66
**Gender** *Male / Female (%)*	25.8% / 74.2%	25.8% / 74.2%	1.00	25.8% / 74.2%	25.8% / 74.2%	1.00
**ABO Blood Group** *O / Non-O (%)*	30.1% / 69.9%	50% / 50%	0.08	25.8% / 74.2%	38.7% / 61.3%	0.23
**Body Mass Index (BMI)** *Mean (SD)*	29.8 (4.8)	27.9 (5.7)	0.10	29.2 (4.8)	30.9 (4.6)	0.09
**Time between DVT** **and study inclusion** *Months- Mean (SD)*	61.4 (47.4)	NA	NA	61.7 (42.8)	60.8 (55.7)	0.94
**Recurrent DVT** *Yes / No (%)*	34.4% / 65.6%	NA	NA	30.6% / 69.4%	41.9% / 58.1%	0.37
**Provoked / Unprovoked** *(%)*	64.5% / 35.5%	NA	NA	69.30% / 30.7%	54.8% / 45.2%	0.18
**Proximal / Distal DVT** *(%)*	90.3% / 9.7%	NA	NA	90.3% / 9.7%	90.3% / 9.7%	1.00
**Use of Anticoagulation** *Yes / No (%)*	68.1% / 31.9%	NA	NA	25.8% / 74.2%	41.9% / 58.1%	0.15
**Use of Statins / Aspirin** *Yes / No (%)*	38.7% / 61.3%	6.5% / 93.5%	**≤0.001**	38.7% / 61.3%	38.7% / 61.3%	1.00

This table shows the clinical characteristics of DVT patients with severe and no/mild PTS. P values were calculated using the Fisher's exact test for parametric analysis, and Mann-Whitney test for Non-parametric analysis. Abbreviations: PTS, post-thrombotic syndrome; HI, healthy individuals; DVT, deep venous thrombosis; SD, standard deviation.

Regarding the use of medication, statin and AAS were present 38.7% in both groups and 6.5% in the HI group. The mean time between the last DVT episode and the inclusion in the study for all DVT patients was 61.4 months. When we evaluated these patients according to PTS severity, there was no significant difference in baseline characteristics between the groups (Table 1).

### Factor VIII and D-dimer

Patients on anticoagulant therapy (29 DVT patients) were excluded from the analyses of D-dimer and FVIII levels, as these markers are affected by the use of anticoagulants. Patients with DVT had higher plasma levels of FVII (186.2 IU/dL vs. 138.2 IU/dL; P ≤ 0.001) and D-dimer (0.60 mg/L vs. 0.17 mg/L; P **≤** 0.001) when compared to HI (Table 2), showing that these patients still presented a hypercoagulability state even after several months of the last thrombotic episode. However, when DVT patients were compared according to the severity of PTS, there was no significant difference between the groups (Table 3). Furthermore, s-TF levels were not detected in the most plasma samples of DVT patients and HI and could not be compared between the groups (data not shown).

**Table 2:** Biomarker levels in DVT patients and controls.

	Total Patients (N = 93)	HI (N = 31)	P
**Factor VIII** (IU / dL)—*Mean (SD)* ¥	186.2 (51.3)	138.2 (44.5)	**≤0.001**
**D-Dimer** *(mg / L)—Mean (SD)* ¥	0.60 (1.13)	0.17 (0.12)	**≤0.001**
**CRP** *(mg/dL)—Mean (SD)*	0.52 (0.53)	0.28 (0.27)	**0.01**
**MCP-1** *(pg/mL)—Mean (SD)*	651.2 (353.6)	657.5 (343.6)	0.82
**TNF-α** *(pg/mL)—Mean (SD)*	11.5 (9.5)	11.3 (5.3)	0.40
**IL-8** *(pg/mL)—Mean (SD)*	14.2 (21.9)	18.6 (22.2)	0.34
**RANTES** *(ng/mL)—Mean (SD)*	76.5 (34.7)	66.4 (25.4)	0.23
**sP-selectin** *(ng/mL)*—*Mean (SD)*	113.8 (49.9)	111.8 (47.2)	0.73
**sICAM-1** *(ng/mL)—Mean (SD)*	60.8 (25.4)	44.9 (9.6)	**≤0.001**
**sVCAM-1** *(ng/mL)—Mean (SD)*	478.1 (140.2)	493.6 (181.9)	0.88
**sE-selectin** *(ng/mL)*—*Mean (SD)*	72.6 (25.6)	64.6 (27.2)	0.22
**MMP-1** *(ng/mL)—Mean (SD)*	7.5 (5.0)	6.3 (3.1)	0.13
**MMP-2** *(ng/mL)—Mean (SD)*	160.9 (45.7)	164.0 (22.3)	0.58
**MMP-3** *(ng/mL)—Mean (SD)*	20.3 (14.2)	20.2 (11.1)	0.84
**MMP-7** (*ng/mL)—Mean (SD)*	9.5 (7.4)	7.9 (2.0)	0.45
**MMP-9** (*ng/mL)—Mean (SD)*	156.3 (84.9)	200.4 (116.6)	**0.01**
**MMP-10** (*pg/mL)—Mean (SD)*	527.3 (344.6)	427.0 (187.5)	0.21
**MMP-12** (*pg/mL)—Mean (SD)*	210.7 (140.7)	206.1 (88.0)	0.59
**MMP-13** (*pg/mL)—Mean (SD)*	294.7 (330.1)	300.1 (278.0)	0.71
**TIMP-1** (*ng/mL*)*—Mean (SD)*	108.6 (33.0)	105.0 (25.5)	0.73
**TIMP-2** (*ng/mL)—Mean (SD)*	62.3 (17.5)	62.3 (20.1)	0.94
**EGF** *(pg/mL)—Mean (SD)*	122.0 (85.5)	135.7 (72.5)	0.15
**PDGF-AA** *(ng/mL)—Mean (SD)*	3.2 (1.0)	3.4 (0.8)	0.36
**PDGF-AB/BB** *(ng/mL)—Mean (SD)*	26.1 (13.7)	25.5 (13.4)	0.76
**TGF-β1** *(ng/mL)—Mean (SD)*	71.8 (48.8)	66.0 (32.5)	0.50
**TGF-β2** *(ng/mL)—Mean (SD)*	4.4 (2.6)	4.1 (1.6)	0.47
**TGF-β3** *(ng/mL) Mean (SD)*	0.3 (0.3)	0.4 (0.3)	0.36

This table shows the biomarker levels in DVT patients and healthy individuals (HI) groups. P values were calculated using the Mann-Whitney test.

¥ Patients under anticoagulant therapy were excluded from these analyses (29 patients excluded).

Abbreviations: DVT, deep venous thrombosis; CRP, C-reactive protein; IL-8, interleukin 8; MCP-1, monocyte chemoattractant protein 1; TNF-α, tumor necrosis factor-alpha; sE-selectin, soluble E-selectin; sP-selectin, soluble P-selectin; sICAM-1, soluble intercellular adhesion molecule 1; sVCAM-1, soluble vascular cell adhesion molecule 1; MMP, matrix metalloproteinase; TIMP, tissue inhibitor of metalloproteinases; PDGF-AA, platelet-derived growth factor-AA; PDGF-AB/BB, platelet-derived growth factor-AB/BB; EGF, epidermal growth factor; TGF-β, transforming growth factor-beta; SD, standard deviation.

**Table 3:** Coagulation marker levels in DVT patients.

	No / Mild PTS (N = 46) ¥	Severe PTS (N = 18) ¥	Mean Difference and 95%CI		P
**Factor VIII** (IU/dL) *Mean (SD)*	176.7 (44.7)	184.2 (66.0)	7.25	-20.07 to 24.86	0.33
**D-Dimer** *(mg/L)* *Mean (SD)*	0.56 (0.99)	0.73 (1.20)	0.18	-0.33 to 0.69	0.48
**sP-selectin** *(ng/mL)* *Mean (SD)*	115.9 (52.9)	108.5 (44.7)	- 6.98	-29.31 to 15.35	0.53

This table shows the coagulation marker levels in patients according to PTS severity. P values were calculated using a general linear model adjusted by Bonferroni correction.

¥ Patients on anticoagulant therapy were excluded from these analyses.

Abbreviations: DVT, deep venous thrombosis; PTS, post-thrombotic syndrome; sP-selectin, soluble P-selectin; SD, standard deviation; CI, confidence interval.

### CRP and inflammatory cytokines

Patients with DVT showed higher levels of CRP (0.52 mg/dL vs. 0.28 mg/dL; P = 0.01) when compared to HI (Table 2). Regarding severity classes of PTS, patients with severe PTS showed significant elevated levels of CRP (0.67 mg/dL) when compared to patients with mild/no PTS (0.42 mg/dL); P = 0.03 (Table 4). Moreover, patients with severe PTS presented decreased MCP-1 levels (554.1 pg/dL) when compared to patients with no/mild PTS (715.7 pg/dL); P = 0.04. There was no statistical difference regarding other inflammatory cytokines when comparing patients and HI, or patients classified according to PTS severity. In addition, IL-1β, IL-6, IL-10, and IL-13 levels were below the limit of detection in most of the samples of DVT patients and controls and could not be analyzed (data not shown).

**Table 4:** Inflammatory marker levels in DVT patients.

	No / Mild PTS (N = 62)	Severe PTS (N = 31)	Mean Difference and 95%CI		P
**CRP** *(mg/dL)* *Mean (SD)*	0.42 (0.50)	0.67 (0.55)	0.25	0.02 to 0.48	**0.03**
**MCP-1** *(pg/mL)* *Mean (SD)*	715.7 (326.1)	554.1 (375.5)	- 161.91	- 314.57 to—9.26	**0.04**
**TNF-α** *(pg/mL)* *Mean (SD)*	12.8 (11.1)	9.5 (5.4)	- 3.31	- 7.38 to 0.77	0.11
**IL-8** *(pg/mL)* *Mean (SD)*	12.1 (13.5)	18.4 (32.5)	6.11	- 3.43 to 15.65	0.21
**RANTES** *(ng/mL) Mean (SD)*	78.2 (30.3)	73.9 (42.9)	- 4.24	- 19.65 to 11.16	0.58

This table shows the inflammatory marker levels in DVT patients according to the PTS severity. P values were calculated using a general linear model adjusted by Bonferroni correction. Abbreviations: DVT, deep venous thrombosis; PTS, post-thrombotic syndrome; CRP, C-reactive protein; IL-8, interleukin 8; MCP-1, monocyte chemoattractant protein 1; TNF-α, tumor necrosis factor-alpha; SD, standard deviation; CI, confidence interval.

### Endothelial dysfunction markers

**Fig 1:**
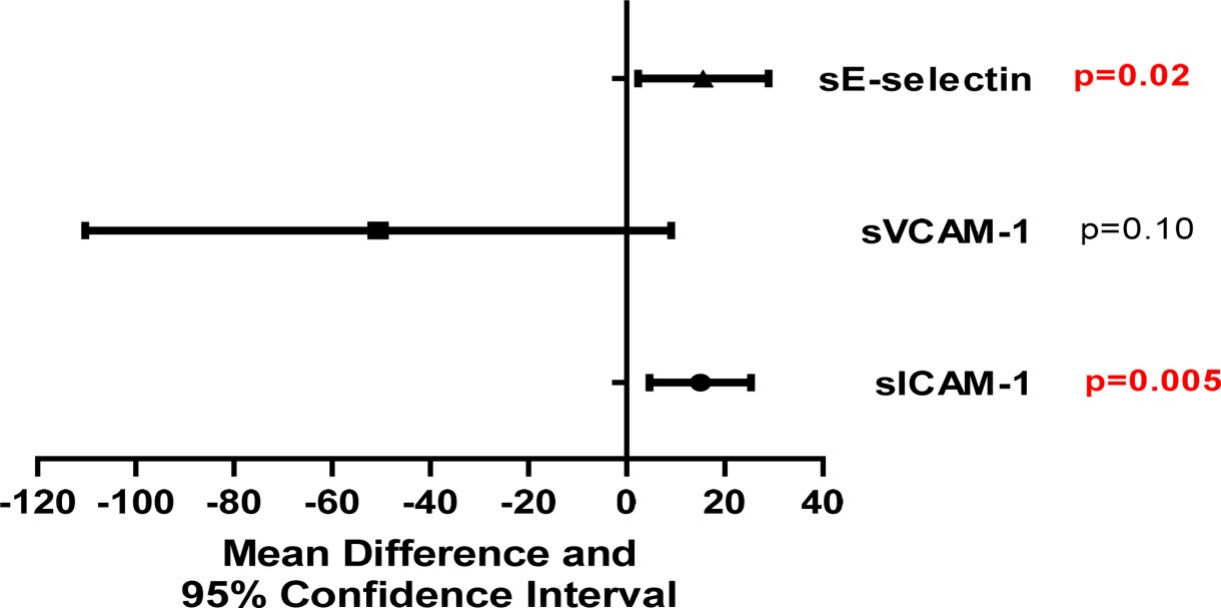
Endothelial dysfunction marker levels in DVT patients. This figure shows the mean difference and 95% confidence interval of endothelial dysfunction marker levels between severe PTS patients and no/mild PTS patients. P values were calculated using a general linear model adjusted by Bonferroni correction. Abbreviations: sICAM-1, soluble intercellular adhesion molecule 1; sVCAM-1, soluble vascular cell adhesion molecule 1; sE-selectin, soluble E-selectin.

Patients with DVT showed higher levels of sICAM-1 (60.8 ng/mL vs. 44.9 ng/mL; P ≤ 0.001) when compared to HI (Table 2). Regarding severity classes of PTS, patients with severe PTS showed significant elevated levels of sICAM-1 (70.5 ng/mL) when compared to patients with mild/no PTS (55.2 ng/mL); P = 0.005. Furthermore, patients with severe PTS showed elevated levels of sE-selectin (80.1 ng/mL), P = 0.02 (Fig 1). There were no significant differences in other endothelial dysfunction markers in patients classified according to PTS severity (Fig 1). In addition, endothelin-1 levels were below the limit of detection in most of the samples of DVT patients and controls and could not be compared (Data not shown).

### Matrix metalloproteinases and their inhibitors

Patients with DVT showed lower levels of MMP-9 (156.3 ng/mL vs. 200.4 ng/mL; P ≤ 0.01) when compared to HI (Table 2). Regarding severity classes of PTS, patients with severe PTS showed decreased levels of MMP-9 (134.5 ng/mL) when compared to controls (169.0 ng/mL); P = 0.04. There was no statistically significant difference between other MMPs in patients classified according to PTS severity *(*Table 5).

**Table 5:** Matrix metalloproteinases and their inhibitors (TIMPs) levels in DVT patients.

	No / Mild PTS (N = 62)	Severe PTS (N = 31)	Mean Difference and 95%CI		P
**MMP-1** *(ng/mL) Mean (SD)*	7.9 (5.0)	6.5 (4.8)	- 1.43	- 3.62 to 0.75	0.19
**MMP-2** *(ng/mL) Mean (SD)*	158.6 (41.9)	167.4 (52.4)	8.27	- 11.74 to 28.28	0.41
**MMP-3** *(ng/mL) Mean (SD)*	21.9 (16.5)	17.4 (8.1)	- 5.54	- 10.57 to 1.48	0.14
**MMP-7** (*ng/mL) Mean (SD)*	10.0 (9.2)	8.5 (2.0)	- 1.65	- 4.79 to 1.48	0.30
**MMP-9** (*ng/mL) Mean (SD)*	169.0 (82.8)	134.5 (87.7)	- 33.53	- 70.40 to—3.34	**0.04**
**MMP-10** (*pg/mL) Mean (SD)*	510.9 (378.6)	558.8 (284.4)	49.80	- 103.93 to 203.54	0.52
**MMP-12** (*pg/mL) Mean (SD)*	202.4 (95.2)	225.8 (203.4)	22.14	- 35.77 to 80.04	0.45
**MMP-13** (*pg/mL) Mean (SD)*	290.1 (260.5)	307.7 (439.2)	15.56	- 131.10 to 162.22	0.83
**TIMP-1** (ng/mL) *Mean (SD)*	109.5 (36.0)	107.7 (28.0)	- 2.24	- 16.79 to 12.31	0.76
**TIMP-2** (*ng/mL) Mean (SD)*	63.2 (17.5)	60.6 (17.9)	- 2.91	- 10.51 to 4.69	0.45

This table shows matrix metalloproteinases and their inhibitors levels in DVT patients according to PTS severity. P values were calculated using a general linear model adjusted by Bonferroni correction. Abbreviations: DVT, deep venous thrombosis; PTS, post-thrombotic syndrome; MMP, matrix metalloproteinase; TIMP, tissue inhibitor of metalloproteinases; SD, standard deviation; CI, confidence interval.

### Tissue remodeling and growth factors

Regarding tissue remodeling and growth factor levels (PDGF-AA, PDGF-AB/BB, EGF, TGF-β1, TGF-β2, TGF-β3), in serum, we observed no significant difference between the groups, according to the severity of PTS. Moreover, FGF-1 and FGF-2 levels were below the limit of detection in most of the samples of DVT patients and HI and could not be compared between the groups (data not shown).

## Discussion

A number of questions remain open regarding the pathophysiology of PTS and clinical manifestations. Pathways like fibrinolysis, chronic inflammation, tissue remodeling, endothelial activation are all involved in this process [6,7].

In this case-control study, we aimed to evaluate the association between PTS and some biological parameters, selected according to possible pathways involved in the pathophysiology of PTS. A panel of blood biomarkers was investigated in patients with and without PTS based on Villalta scale [18]. PTS is manifested up to 2 years after an acute episode of DVT, and in this study, the mean time between the most recent DVT episode and study inclusion was 61 months. Moreover, we observed no significant difference in mean time between the most recent episode of DVT and the inclusion in this study according to PTS severity groups. This is a relevant point in order to exclude this important confounding factor.

In the past years, several studies have investigated the association between D-dimer levels and PTS. However, there has been a significant heterogeneity between studies regarding time point of D-dimer evaluation after DVT, population diversity and measurement methodology. D-dimer levels were analyzed in the acute or subacute phase of the DVT episode, and a few of them found a significant association between D-dimer levels and PTS [21–23], whereas other studies found no association [24–28]. Corroborating our results, four previous studies demonstrated no significant association between D-dimer and PTS [29–31] during the chronic phase (more than 12 months post DVT). Our results suggest that a hypercoagulability can be present up to several months after acute DVT episode, however with no association with PTS.

We showed increased CRP levels in patients when compared to HI and according to PTS severity, suggesting that there is a persistent inflammatory process in those patients. Previous studies showed an association between increased levels of CRP and PTS [19,26,32,33]. However, in these studies, blood sampling was collected a few months after DVT and could possibly still reflect residual inflammatory activity from the acute thrombotic episode. A recent study by Bouman et al [30] with a similar mean time between the last DVT episode and study inclusion, showed no association between CRP levels and PTS; they, however, only compared CRP levels in patients with and without PTS and did not consider PTS severity. Indeed, they only included 4 patients (13%) with severe PTS. Our results indicate a persistent inflammatory response more evident in patients with severe PTS and strengthen the idea that inflammation plays an important role in the maintenance of the signs and symptoms of severe PTS. These findings raise the hypothesis that elevated CRP levels during the chronic phase of DVT are probably more related to PTS severity than to the acute thrombotic episode.

Coagulation, inflammation and endothelial dysfunction are important processes involved in DVT and PTS, and they are closely linked. Valves and vein wall are damaged after the thrombotic episode, as a consequence of the inflammatory process in response to thrombus growth, organization, and vein recanalization (8). Moreover, pro-inflammatory cytokines and thrombin induce expression of endothelial cell adhesion molecules (CAMs) and their soluble forms which act to facilitate the adhesion of leukocytes to the endothelium [34]. Thus, all of these activities upon the propagation of the inflammatory state. ICAM-1 and E-selectin are adhesion molecules involved in leukocyte-endothelium interactions, facilitating leukocyte endothelial transmigration. Endothelial cells can be activated by inflammatory cytokines and thrombin that preclude an upregulation of membrane-bound as well as soluble adhesion molecules [35,36].

Previous studies showed elevated sICAM-1 levels in patients with PTS [19,37], analyzed a few months after DVT episodes and did not evaluate PTS grade. Our results demonstrated increased sICAM-1 levels in severe PTS when compared to others. We also showed that elevated sE-selectin levels were associated with PTS grade. Two previous studies evaluated sE-selectin levels during the acute phase of DVT and showed no association between DVT and increased sE-selectin levels [20,38]. Our findings are interesting considering that we demonstrated endothelial dysfunction even a long time after the acute DVT episode, and this seems to be associated with PTS severity. This could promote leukocytes adhesion perpetuating the local inflammatory process worsening the signs and symptoms of PTS.

MMPs are a group of endopeptidases with the capacity of cleaving several components of the extracellular matrix (ECM), such as collagen, elastin, gelatins, and others. Leukocytes are a major source of MMPs, however, fibroblasts, endothelial cells, and smooth muscle cells also express MMPs [39]. MMPs play a role in many biological processes including tissue remodeling and growth, wound healing, immune response, cell proliferation, migration, differentiation, and apoptosis [40]. Studies suggest that alterations in MMPs activity could result in pathological changes in the vein wall and valves leading to vascular diseases, such as varicose veins, skin ulcers and chronic venous insufficiency [41].

MMP-9 is important in vascular remodeling and thrombus resolution [42–44]. Studies using murine DVT models with deletion or inhibition of MMP-9 observed impaired thrombus resolution and significantly less vein wall fibrosis and inflammation (14, 16, 44). De Franciscis et al (2015) demonstrated increased MMP-9 levels in the acute phase of DVT when compared to healthy controls. However, after a follow-up of 18 months, DVT patients presented similar MMP-9 levels to controls [45]. Our study evaluated patients with a mean time of 61 months after the last DVT episode and we found that DVT patients presented decreased MMP-9 levels when compared to no/mild PTS. MMP-9 is a pro-angiogenic factor and has been associated with tumor angiogenesis and progression [46,47]. Our hypothesis is that the decreased MMP-9 levels found in our DVT patients, more expressive in patients with severe PTS, may contribute to a poor vascular repair process, and consequently to the clinical manifestations of PTS. Noteworthy, TIMP-2, regulator of MMP-9, did not follow the aforementioned changes. This could have happened due to the fact that TIMP-2 expression can be regulated by a wide range of agents, including α2-macroglobulin, growth factors, cytokines, and other soluble mediators. Thus, it is important not to generalize the findings to other MMPs and TIMPs, especially as vein remodeling is a dynamic process and the activities of MMPs may vary during the course of the disease [40,48–50].

MCP-1 is a potent chemoattractant for monocytes/macrophages, and MCP-1 production can be seen in many different cells such as astrocytes, smooth muscle cells, fibroblasts, monocytes/ macrophages, and endothelial cells in response to inflammation. MCP-1 can induce the migration and infiltration of macrophages/monocytes and plays an important role in inflammation and angiogenesis pathways [51,52]. Previous studies suggest that MCP-1 has an important role in DVT resolution and recanalization but the mechanisms involved in this process remain unknown [53]. The direct action of MCP-1 on endothelial cells or macrophages stimulation inducing vascular channels or VEGF expression can explain its angiogenic properties. Herein, we hypothesized that as MMP-9, the decreased MCP-1 levels found in our DVT patients, more expressive in patients with severe PTS, may also impair vascular repair process, and consequently contribute to the signs and symptoms of PTS.

With our study design selecting only extreme levels of PTS (mild and severe), we were able to demonstrate that PTS severity leads to an important difference between DVT’s biomarkers. In addition, since clinical symptoms among the groups with mild PTS and no PTS seem to be very close, both groups were combined. In addition, previous studies only included a small number of patients with severe PTS, and this is an important contribution of our study, as a high number of patients with this rare PTS classification were included.

Thus far, strengths of the present study are the measurement of a comprehensive panel of coagulation, inflammatory, endothelial dysfunction, tissue remodeling and growth factors, and a careful clinical evaluation of PTS, in a well-defined population. The inclusion of a high number of patients with severe PTS is of particular relevance. Finally, to avoid bias by multiple parameters evaluated, we performed a careful statistical analysis using a general linear model adjusted by Bonferroni correction. This study, as well as the aforementioned, has limitations. Several biomarkers could not be evaluated due to the low sensitivity of the method. Furthermore, blood sampling performed once, after a long period of time after the acute episode did not allow us to consider these biomarkers as the cause or consequence of PTS. Additionally, as this study was an investigative pilot study, the results should be validated in a larger group of patients, classified by severity of PTS.

Furthermore, according to the literature, neutrophil extracellular traps (NETs) appear to also be capable of inducing CAMS expression, such as V-CAM-1 and I-CAM1, through endothelial cells. Since NETs and endothelial cells have been recently associated with DVT through the role of inflammation, one of our major limitations was the absence of investigation and correlation between NETS, I-CAM and PTS severity [54,55].

In general, a significant change in levels was detected only in CRP, sICAM-1, sE-selectin, MMP-9, and MCP-1 markers. The absence of significance alterations in the rest could be explained by the sample size or because cytokines and MMPs are part of a complex system that have different activation pathways and higher levels of a determined cytokine may not necessarily effect inflammation of other cytokines n [40].

## Conclusions

In conclusion, we demonstrated that DVT patients present an inflammatory status, endothelial dysfunction and altered proteolysis MMPs activity, even a long time after the acute thrombotic episode. Indeed, all these alterations were associated with severe PTS, suggesting a possible relation with the maintenance and worsening of PTS severity. This finding highlights the importance of the evaluation of PTS severity in DVT studies. Moreover, based on previous studies and on our results, from all the biomarkers evaluated, sICAM-1 levels appear to be the parameter most strongly associated with PTS, and this might be a potential target for further research.

## Supporting information

S1 Fig Clinical and laboratory data. Data description of all patients recruited.
(PDF)
