# Supplementary material for: Increased inflammation and endothelial markers in patients with late severe post-thrombotic syndrome
Source: PLoS One. 2020 Jan 16;15(1):e0227150. doi: 10.1371/journal.pone.0227150 (PMC6964981; doi:10.1371/journal.pone.0227150)
Supplement: S1 Fig — Data description of all patients recruited. (PDF) [file pone.0227150.s001.pdf]

Age	Gender	Number of episodes	Characteristic of ocurrance	SAF
38	Fem	1	Provoked	No
63	Fem	3	Unprovoked	No
60	Fem	1	Unprovoked	No
30	Male	1	Unprovoked	No
54	Fem	1	Provoked	No
50	Fem	2	Provoked	No
24	Male	2	Provoked	No
31	Fem	2	Provoked	No
40	Fem	1	Provoked	Yes
46	Fem	1	Provoked	No
59	Fem	2	Unprovoked	No
60	Fem	2	Unprovoked	No
70	Fem	1	Provoked	No
41	Male	1	Provoked	No
58	Fem	2	Unprovoked	No
51	Male	2	Provoked	No
52	Male	2	Unprovoked	No
43	Fem	1	Provoked	
58	Fem	2	Unprovoked	No
57	Male	1	Unprovoked	No
55	Fem	1	Provoked	No
50	Fem	3	Provoked	Yes
61	Fem	1	Unprovoked	No
59	Fem	1	Provoked	No
45	Fem	2	Provoked	No
47	Fem	1	Provoked	No
36	Fem	3	Unprovoked	No
60	Male	2	Provoked	No
65	Fem	1	Provoked	No
51	Fem	1	Unprovoked	No
34	Fem	1	Unprovoked	No
65	Male	1	Unprovoked	No

Characteristic of re-ocurrance	Time between last DVT episode and SPT evaluation (months)	Anticoagulation	BMI	Abdominal circuference
NA	82	No	29,14	97
Unprovoked	14	Yes	39,54	130
NA	96	No	42,72	117
Unprovoked	47	Yes	39,6	130
NA	93	No	38,13	119
Provoked	20	No	34,8	111
Provoked	68	Yes	27,46	107
Unprovoked	71	No	30	101
NA	130	Yes	35,32	116
NA	57	No	33,78	100
Provoked	60	Yes	31,52	95
Unprovoked	9	Yes	31,09	108
NA	49	No	30,7	94
NA	50	No	29,06	100
Unprovoked	8	No	29,34	105
Provoked	47	No	30,07	103
Unprovoked	276	Yes	28,43	103
NA	52	No	21,78	74
Provoked	15	Yes	31,61	110
NA	19	No	29,41	110
NA	62	No	25,19	98
Provoked	14	Yes	32,42	104
NA	130	No	27,85	95
NA	124	No	26,37	104
Provoked	48	Yes	27,48	99
NA	80	Yes	24,66	90
Unprovoked (2) e Provoked (1)	4	Yes	32,67	113
Provoked	2	Yes	31,23	117
NA	21	No	24,51	84
NA	60	No	31,58	93
NA	4	Yes	30,45	100
NA	135	No	29,76	106

Obesity	Villalta Scale	Ulcer	D-Dimer	FVIII	FVW	EGF
No	19	No	0,38	213,6	156,4	3,8
Yes	16	No	0,71	278,4	285,2	17,6
Yes	19	No	0,63	261,1	161,9	115,45
Yes	15	No	1,32	193,8	161,3	140,19
Yes	15	No	0,33	162,6	114,1	109,45
Yes	15	No	0,17	157	117,7	154,06
No	21	Yes	0,17	210	154,8	144,13
Yes	17	No	0,28	133,9	122,3	285,35
Yes	20	No	0,17		122,5	20,21
Yes	20	No	0,5	234	216,4	72,68
Yes	18	No	0,38	130	102,6	247,74
Yes	15	No	0,17	176,7	154,5	75,63
Yes	16	No	0,39	207,5	144,8	113,46
No	20	Yes	4,52	121,1	113,5	110,06
No	16	No	0,33	124,8	124,6	141,44
Yes	13	Yes	0,54	150,2	120,7	178,18
No	13	Yes	0,27			106,23
No	15	No	0,1	165,7	190,5	174,06
Yes	18	No	0,22	196,1	136,8	212,86
No	14	Yes	0,52	385,5	152,4	3,8
No	16	No	0,26	216,8	156,6	3,8
Yes	19	No	0,08	212,6	137,7	131,77
No	14	Yes	0,3	174,6	135	161,74
No	22	No	1,25	237,6	368	56,5
No	15	No	1,05	176,7	145,9	134,39
No	15	No	0,26	141,3	125	51,47
Yes	19	No	0,23	229	198,6	160,86
Yes	19	No	6,04	126,7	132,8	40,63
No	23	No	0,52	196,8	168	3,8
Yes	15	Yes	0,14	193,7	139,4	281,61
Yes	22	No	0,06	158,2	111,6	29,57
No	16	No	0,26	97,7	95	33,74

Endotelin-1	FGF-1	VEGF-C	VEGF-D	VEGF (Total?)	MCP-1
32,61	13	73,51	9	10	5,6
3	13	102,79	9	4359,53	291,54
3	13	151,52	90,55	94,19	199,42
3	13	204,37	347,05	72,96	294,42
41,2	36,67	362,77	55,19	662,33	4
3	13	127,92	380,53	10	387,03
3	13	184,29	9	47,97	15,39
3	13	78,66	88,02	10	1257,08
3	13	189,36	9	432,01	809,67
3	13	71,77	150,48	10	634,95
23,49	13	179,17	9	10	452,95
3	13	205,61	58,97	10	885,26
3	13	91,81	9	10	392,82
14,14	13	31,54	9	105,31	582,12
3	13	93,41	221,65	10	646,06
3	13	155,56	114,68	171,02	464,1
19,32	13	55,22	300,5	10	609,88
3	13	114,85	450,78	10	709,98
66,83	13	186,83	242,55	179,64	1003,07
3	13	73,51	239,94	10	255,82
3	13	156,89	149,2	801,98	894,77
3	13	125,06	36,3	171,02	747,52
3	13	113,36	9	408,71	1707,26
26,27	13	98,76	616,16	262,42	744,34
3	13	55,28	9	10	583,92
3	13	56,28	463,91	72,96	624,53
2,32	5,32	110,9	118,9	89,13	634,14
3	13	64,2	224,88	225,68	675,05
3	13	46,2	53,89	32,67	524,21
3	13	85,5	28,4	83,91	666,37
3	13	68,12	9	10	13,61
		64,2	135,81	122,19	15,96

TNF-Alfa	IL-1b	IL-6	IL-8	IL-10
3	0,3	3	3	3
7,02	0,3	104,02	108,09	3
4,94	0,91	3	13,52	3
9,97	0,3	3	2,51	3
3	1,02	3	3,36	3
8,47	0,3	3	3,06	3
3,43	2,18	10,18	3	21,75
16	0,3	3	4,89	3
19,21	51,72	25,8	67,45	7,49
12,06	0,3	3	13,97	3
8,52	0,3	3	11,69	26,44
8,78	0,3	3	10,21	3
4,67	0,3	3	6,56	3
10,53	0,3	3	11,91	3
13,06	0,3	3	3,71	3
9,5	0,3	3	4,34	3
7,66	0,3	3	3	3
6,23	0,3	3	8,46	3
17,51	0,3	3	5,68	3
9,82	0,3	3	4,1	3
25,05	0,3	7,98	22,08	3
13,56	0,3	3	15,77	3
15,61	0,3	3	154,42	3
10,6	0,3	3	13,39	3
9,44	0,3	3	13,71	3
3	0,3	3	25,66	3
7,76	0,3	3	11,53	3
13,43	0,97	3	24,62	3
4,67	0,8	3	1	3
12,94	0,3	3	8,65	3
2,15	0,3	3	3	3
3	0,3	3	2,93	3

IL-13	FGF-2	MMP-1	MMP-2	MMP-3
3	3	2527,1	150530,35	8831,72
103,23	40,74	1619,25	195287,65	27458,46
3	15,14	21095,13	231575,28	7468,77
3	26,87	7851,14	131300,37	14931,37
3	19,24	7726,8	164917,2	8152,23
3	9,16	23068,31	150282,72	20050,76
3	25,64	10363,05	134395,52	14248,18
3	12,24	3290,29	116519,03	12418,56
457,42	109,47	9022,8	123718,48	14727,25
3	3	5026,29	161057,47	12398,26
3	3	6398,49	150777,85	23154,26
3	3	7835,59	259104,46	14936,48
3	3	6321,75	105014,75	17418,87
3	60,43	5877,81	148794,09	22269,18
3	3	4754,31	195969,92	11087,92
3	3	9983,35	187270,13	27787,25
3	3	5893,09	140770,03	33708,01
3	3	5481,49	185653,45	27571,8
3	88,03	7308,11	156189,97	9906,5
3	3	977,93	145925,05	44751,2
52,92	781,48	1755,97	124631,69	23599,19
3	80,77	7083,41	178570,29	17144,39
3	3	9007,11	151766,54	14365,33
3	64,82	6598,27	392565,93	10579,25
3	3	3379,02	171602,28	15980,51
3	3	3438,25	184264,12	18344,72
3	3	3839,46	129483,75	14125,99
3	39,63	6798,42	174913,22	16963,66
3	76,11	2895,74	182612,71	16239,54
3	5,78	8019,34	140116,18	15435,72
3	30,48	1090,94	134228,94	8722,66
3	3	3054,32	156189,97	13342,83

MMP-7	MMP-9	MMP-10	MMP-12	MMP-13
6158,37	84856,88	329,35	32,99	10
7285,23	83923,91	376,64	112,52	178,33
9867,65	72187,48	623,31	106,7	243,79
6158,37	126986,16	482,13	322,33	596,12
7374,41	83032,9	363,26	233,5	718,84
9522,75	197730,23	452,41	234,84	420,49
5331,19	109204,53	509,32	191,34	305,24
9695,38	321650,89	794,65	126,93	10
9089,52	114470,01	718,45	174,79	58,55
9002,58	89666,34	699,41	194,09	339,06
13247,14	127510,09	376,64	199,57	475,41
6882,21	47249,11	357,72	213,2	412,52
10253,95	61993,64	338,8	94,95	58,55
7818,41	92128,08	433,46	988,72	83,23
11233,55	328475,59	1329,71	158,07	158,59
5884,27	68660,11	547,29	144	225,57
10851,48	271310,34	585,29	202,3	83,23
5792,55	92981,66	851,85	112,52	10
6792,26	115097,01	357,72	196,83	188,01
10998,91	22198,02	572,5	881,94	2446,35
4297,19	243782,96	271,57	80,43	235,49
9821,47	154694,77	590,41	272,77	274,96
10296,76	81784,79	547,29	213,2	396,47
9176,36	64369,79	509,32	207,76	288,01
9996,61	93957,79	509,32	90,51	188,01
8391,08	262055,47	1540,54	42,81	10
10039,56	146407,96	547,29	205,03	690,39
9089,52	71113,97	282,13	402,58	452,06
7641,72	50739,71	341,68	275,54	24,14
8592,58	347696,82	302,08	58,72	10
5754,34	128215,86	412,27	245,12	10
8566,33	149755,35	928,16	207,76	243,79

PDGF-AA	PDGF-AB/BB	RANTES	PAI-1 (Total)	sP-selectina
1862	31163,71	47916,05	251806,14	6,77
2510,99	34039,87	85514,55	290285,34	81,65
4023,64	40971,16	125418,57	368656,89	97,19
4452,17	34764,37	80435,97	415145,05	157,89
3663,11	37549,79	83778,11	306135,52	64,81
4218,09	26410,43	175824,48	333884,63	138,89
3401,63	16854,38	130983,2	312742,48	81,65
3782,93	16675,66	50724,2	351212,23	132,33
4417,36	33928,2	109921,23	374675,44	104,58
3815,77	28260,07	45618,85	197432,02	85,64
3267,9	44774,39	75922,58	326738,05	108,2
3325,51	16794,78	93800,5	260968,15	138,89
2773,09	25208,26	51923,86	298566,69	118,78
1864,79	13333,17	27400,09	218207,54	111,78
3850,23	22765,86	101131,06	305891,08	104,58
2882,07	18716,37	39254,8	232938,86	95,69
2890,94	15373,3	33644,94	395318,5	223,74
1194,97	22331,22	22979,83	191831,4	55,7
2714,48	8351,07	163786,57	313722,49	64,81
1000	865,05	2761,36	181807,19	204,22
3226,79	20458,85	72199,01	238275,91	147,72
4434,45	26809,19	83745,06	521744	84,93
3394	26537,43	60337,47	278385,21	142,13
3954,06	41106,17	156403,08	380440,43	125,63
2965,04	14844,86	73444,05	363036,3	193,39
3252,77	16378,37	65165,25	284451,72	97,19
3872,2	42324,03	97121,77	429497,84	170,05
3012,64	28837,61	25027,34	267732,18	111,78
2010,02	15606,13	52470,31	218913,65	19,46
5518,22	26982,99	75771,59	431560,9	130,66
3183,79	19613,66	34326,31	260650,02	97,47
2705,7	18474,59	21466,11	266281,78	84,93

sICAM-1	sVCAM-1	TGF-Beta - 1	TGF-Beta - 2
47,64	336,59	97838,51	6272,05
41,61	304,55	59692,4	3928,69
55,39	453,71	56382,77	1268,49
84,52	389,09	1197,72	907,3
59,21	559,86	4637,91	1268,49
86,72	401,82	126612,11	8874,34
56,07	363,75	152643,56	7053,06
51,56	253,37	143110,12	4819,72
48,03	420,44	128882,83	11173,46
66,23	518,57	77323,56	4819,72
59,57	460,66	107505,63	7165,44
49,42	435,47		
67,08	363,18	16353,44	2949,39
179,23	275,32	39232,82	2167,05
48,24	382,08	82793,08	5971,31
51,54	465,59	71248,72	5787,39
70,69	368,17	50097,97	3579,96
59,39	411,5	31683,85	2813,48
53,61	283,31	40123,81	4041,11
171,42	506,02		
55,65	546,58	56141,02	3499,82
40,96	553,15	82261,92	3737,22
46,09	442,54	56636,93	5474,39
62,12	574,4	42903,16	2478,94
49,1	485,26	54579,49	2813,48
67,43	443,14	21541,33	2720,56
61,02	356,87	20	797,27
127,45	486,09	74013,47	4579,71
65,64	453,43	13662,69	5249,96
138,7	688,33	168698,73	7109,35
71,41	614,16	11632,95	1346,63
64,62	735,04	46777,18	2949,39

TGF-Beta - 3	TIMP-1	TIMP-2	PCR	HBGLI	VHS
20	77398,23	51776,05	0,6	5,2	17
20	109480,2	67253,7	2,16	5,9	64
20	131145,82	59171,74	0,11	6	14
20	169100,31	52790,01	0,81	5,5	6
20	120793,86	50759,95	0,51	7,3	2
20	91369,25	41749,05	0,7	6,1	13
20	80864,76	39517,68	1	5,6	3
461,11	119635,61	54138,91	0,4	5,7	14
867,79	70034,13	39649,52	0,25	5,9	20
680	83947,53	50759,95	0,37	7,6	23
680	119279,88	61468,08	0,19	5,5	11
439,9	128488,11	62218,54	0,13	6,2	8
439,9	137100,73	66671,77	0,24	5,6	6
439,9	132122,79	55400,62	0,6	5	3
569,85	112746,92	43485,45	1,52	5,9	2
439,9	91096,89	47739,8	0,32	9,7	9
569,85	86618,06	44263,29	1,43		9
867,79	132757,44	61968,44	0,04	5	5
439,9	66695,04	43398,89	0,6	6,9	25
20	81479,61	54812,15	1,25	6,4	45
20	85698,85	42878,98	0,21	6,2	8
680	86246,3	63843,06	0,28	6,1	10
20	104296,65	39473,71	0,46	5,6	46
439,9	192779,34	121751,65	0,22	6,3	2
439,9	114614,6	85993,68	0,36	5,3	5
20	74780,45	75815,1	0,12	5,2	2
20	110463,2	80851,9	1,11	6,9	40
20	128395,16	88569,05	0,31	5,5	6
289,62	109733,18	75524,66	1,11		
20	103602,74	73879	0,9		
20	92311,13	71991,49	1,54		
20	101262,16	69280,99	1,74	9	12
